# Construction of an RNA modification-related gene predictive model associated with prognosis and immunity in gastric cancer

**DOI:** 10.1186/s12859-023-05283-3

**Published:** 2023-04-15

**Authors:** Airexiati Tuhongjiang, Feng Wang, Chengrong Zhang, Sisi Pang, Yujiang Qu, Bo Feng, Gulimire Amuti

**Affiliations:** Department of Day Surgery, People’s Hospital of Xinjiang Uygur Autonomous Region, Ürümqi, China

**Keywords:** RNA modification, Gastric cancer, Prognosis, Bioinformatics, Epigenomic

## Abstract

**Background:**

Gastric cancer (GC) is one of the most common causes of cancer-related fatalities worldwide, and its progression is associated with RNA modifications. Here, using RNA modification-related genes (RNAMRGs), we aimed to construct a prognostic model for patients with GC.

**Methods:**

Based on RNAMRGs, RNA modification scores (RNAMSs) were obtained for GC samples from The Cancer Genome Atlas and were divided into high- and low-RNAMS groups. Differential analysis and weighted correlation network analysis were performed for the differential expressed genes (DEGs) to obtain the key genes. Next, univariate Cox regression, least absolute shrinkage and selection operator, and multivariate Cox regression analyses were performed to obtain the model. According to the model risk score, samples were divided into high- and low-risk groups. Enrichment analysis and immunoassays were performed for the DEGs in these groups. Four external datasets from Gene Expression Omnibus data base were used to test the accuracy of the predictive model.

**Results:**

We identified *SELP* and *CST2* as key DEGs, which were used to generate the predictive model. The high-risk group had a worse prognosis compared to the low-risk group (*p* < 0.05). Enrichment analysis and immunoassays revealed that 144 DEGs related to immune cell infiltration were associated with the Wnt signaling pathway and included hub genes such as *ELN*. Overall mutation levels, tumor mutation burden, and microsatellite instability were lower, but tumor immune dysfunction and exclusion scores were greater (*p* < 0.05) in the high-risk group than in the low-risk group. The validation results showed that the prediction model score can accurately predict the prognosis of GC patients. Finally, a nomogram was constructed using the risk score combined with the clinicopathological characteristics of patients with GC.

**Conclusion:**

This risk score from the prediction model related to the tumor microenvironment and immunotherapy could accurately predict the overall survival of GC patients.

**Supplementary Information:**

The online version contains supplementary material available at 10.1186/s12859-023-05283-3.

## Background

Gastric cancer (GC) is the fifth most diagnosed malignancy worldwide [1]. The most common pathological type of GC is stomach adenocarcinoma (STAD), which originates from the epithelial cells of the mucosa [2]. Surgery is the cornerstone treatment for GC [3], and survival rates have been improved using chemotherapy [4]. Although many individuals with GC have benefited from the recent development of tailored treatment options, such as immunological, targeted, and combination therapies [5], the mortality rate of GC remains high, making it the third most common cause of cancer-related death [6]. It is therefore important to identify novel prognostic markers and therapeutic targets for patients with GC.

Over 170 different types of modifications for coding and non-coding RNA (ncRNA) have been identified, including N6-methyladenosine (m6A), 5-methylcytosine (m5C), and N1-methyladenosine (m1A) [7]. The methyltransferases, demethylases, and binding proteins, together known as “writers,” “erasers,” and “readers,” can control the dynamics and reversibility of RNA epigenetic modifications [8, 9]. Accumulating evidence suggests that the aberrant expression of RNA modifications is associated with cell survival, proliferation, self-renewal, differentiation, stress adaptation, invasion, and resistance to therapy, all of which are hallmarks of cancer [10–17]. Abnormal post-transcriptional alterations contribute toward cancer cell migration, self-renewal, proliferation, and survival [18] and thus are promising therapeutic targets for cancer. The most prevalently distributed RNA post-transcriptional modification is m6A [19]. It has been reported that the m6A writers METTL3 and METTL14 have an impact on GC progression [20–22], the expression of METTL3 is associated with GC prognosis [21]. Therefore, studying the relationship between RNA modification-related genes (RNAMRGs) and GC would facilitate exploration of potential prognostic markers and therapeutic targets.

**Fig. 1:**
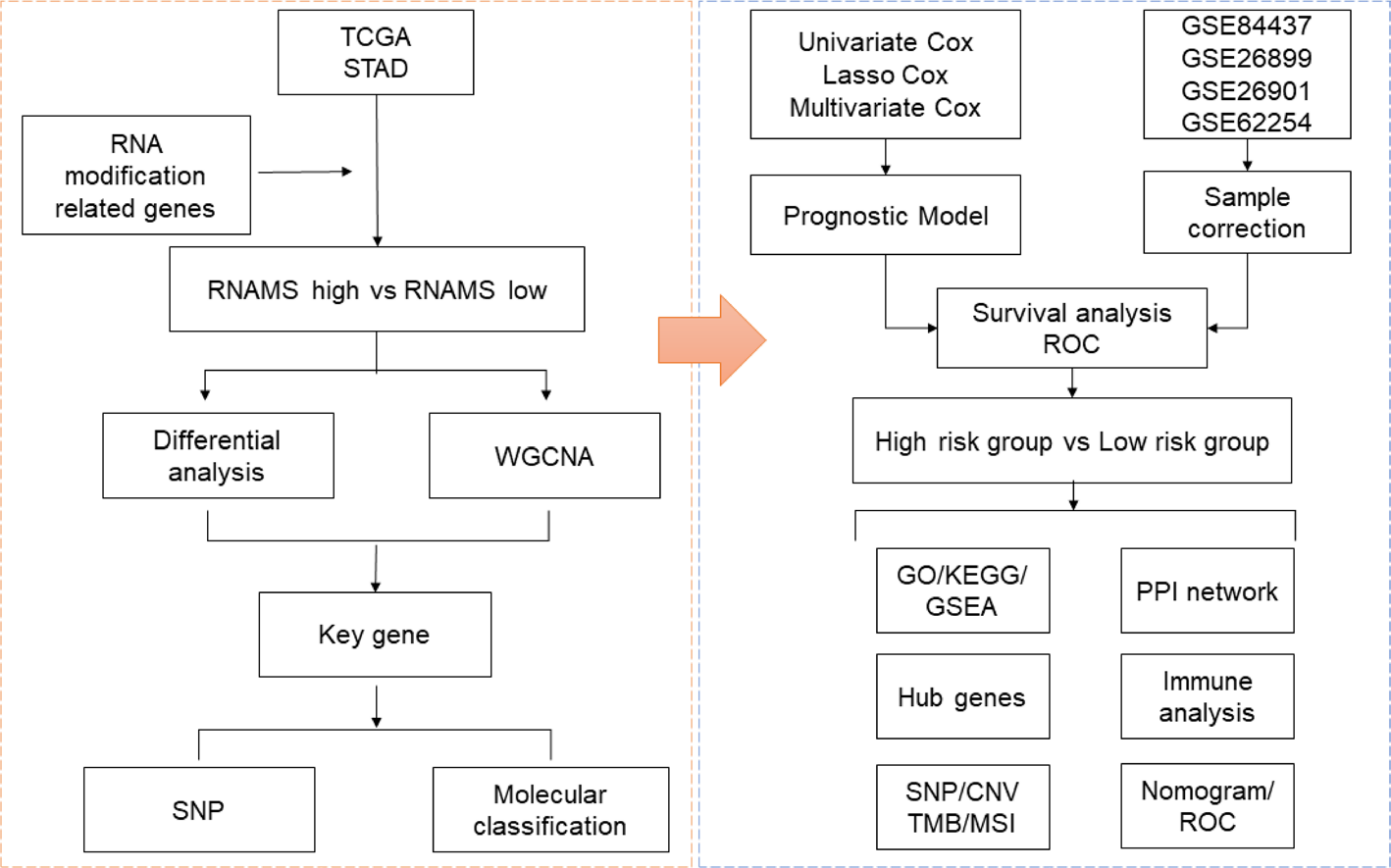
Study flow chart

In this study, we identified 48 RNAMRGs associated with m6A, m5C, and m1A. Similar to bioinformatics methods of some studies [23–26], we used The Cancer Genome Atlas (TCGA) data to screen prognosis-related genes and built a predictive model according to the study flowchart shown in Fig. 1 to explore whether RNAMRGs were associated with GC prognosis.

## Methods

### Data collection

Expression profiles and clinical data of patients with STAD were downloaded from TCGA. GC samples with an overall survival (OS) > 0 were retained, leaving a total of 328 samples (Table 1). "Masked Somatic Mutation" data were downloaded and analyzed. The tumor mutational burden (TMB) and microsatellite instability (MSI) were obtained. “Masked Copy Number Segment” data were used as copy number variation (CNV) data for 440 patients with STAD, who were divided into high- and low-risk groups. Segments were analyzed using GenePattern (https://cloud.genepattern.org) for GISTIC 2.0 [27] with default settings and a confidence level of 0.99. The STAD datasets GSE26899 (93 samples) [28], GSE26901 (109 samples) [28], GSE84437 (357 samples) [29], and GSE62254 (300 samples) [30] were downloaded from Gene Expression Omnibus (GEO) [31], and 48 RNAMRGs (Additional file 1: Table S1) were extracted from an article [32].

**Table 1:** Characteristics of patients with gastric cancer in TCGA-STAD data

	Alive	Dead	Total
	258	70	328
*Age (years)*			
Mean	64.7	67.4	65.2
Median	66	68.5	67
> 67	116 (45%)	42 (60%)	158 (48.2%)
≤ 67	142 (54%)	28 (40%)	170 (51.8%)
*Gender*			
Female	101 (39.1%)	14 (20%)	115 (35.1%)
Male	157 (60.9%)	56 (80%)	213 (64.9%)
*T stage*			
T1	14 (5.4%)	1 (1.4%)	15 (4.6%)
T2	54 (20.9%)	12 (17.2%)	66 (20.1%)
T3	116 (45%)	36 (51.4%)	152 (46.3%)
T4	74 (28.7%)	21 (30%)	95 (29%)
TX	0 (0%)	0 (0%)	0 (0%)
*N stage*			
N0	85 (33%)	14 (20%)	99 (30.2%)
N1	67 (26%)	16 (22.9%)	83 (25.3%)
N2	53 (20.5%)	16 (22.9%)	69 (21%)
N3	47 (18.2%)	22 (31.3%)	69 (21%)
NX	6 (2.3%)	2 (2.9%)	8 (2.5%)
*M stage*			
M0	235 (91.1%)	56(80%)	291 (88.7%)
M1	11 (4.3%)	11(15.7%)	22 (6.7%)
MX	12 (4.6%)	3(4.3%)	15 (4.6%)
*Stage*			
I	34 (13.2%)	9 (12.9%)	43 (13.1%)
II	96 (37.2%)	8 (11.4%)	104 (31.7%)
III	108 (41.9%)	31 (44.3%)	139 (42.4%)
IV	15 (5.8%)	18 (25.7%)	33 (10.1%)
X	5 (1.9%)	4 (5.7%)	9 (2.7%)

### RNA modification score (RNAMS) analysis

The RNAMS was calculated for TCGA samples based on the RNAMRGs using the “ssGSEA” algorithm. Samples were divided into high- and low-RNAMS groups, and differentially expressed genes (DEGs) were screened using adj. *p* < 0.01 and |log fold change (FC)|> 1.

Weighted gene correlation network analysis (WGCNA) was performed using TCGA data as the input, and 0.85 was calculated as the optimal soft threshold by the “pickSoftTreshold” function. With 200 as the minimum number of genes per module, gene modules were dynamically assigned to identify genes. We screened four modules with the strongest positive and negative correlations with the high-RNAMS group. Genes with module membership > 0.5 and significance > 0.2 were screened as key gene modules. DEGs in the high- and low-RNAMS groups were intersected with key gene modules to obtain key genes.

Consensus clustering was performed using TCGA data and the key genes to improve the differentiation between different GC subtypes. The number of clusters was set between 2 and 8, and 80% of all samples were drawn in 1000 repetitions with clusterAlg = “pam” and distance = “spearman.”

### Risk prediction model construction

Univariate Cox regression analysis was used to calculate the association between the expression of each key gene and OS. The least absolute shrinkage and selection operator (LASSO) algorithm was then used to eliminate multicollinearity and screen for significant variables. Following multivariate Cox regression analysis, final screening was performed using stepwise regression. The risk score formula was calculated by considering the expression of optimized genes and the multivariate Cox regression coefficients, as follows:

$$Risk score = \mathop \sum \limits_{i} Coefficient\left( {gene_{i} } \right) \times mRNA expression\left( {gene_{i} } \right)$$

TCGA patients were divided into high- and low-risk groups based on the calculated optimal risk score cutoff. Kaplan–Meier analysis and log-rank tests were performed, and time-dependent receiver operating characteristic (ROC) curves were used to assess survival prediction by calculating the area under curve (AUC).

GSE26901, GSE26899, GSE84437 and GSE62254 were used as validation datasets. After calculating risk scores using the formula above, data were grouped according to the optimal risk score cutoff and subjected to Kaplan–Meier analysis and log-rank tests.

### Risk score analysis

Differential analysis was performed based on the high- and low-risk groups from TCGA data, with adj. *p* < 0.01 and |logFC|> 1.5 as screening thresholds. Gene Ontology (GO) analysis [33] and Kyoto Encyclopedia of Genes and Genomes (KEGG) pathway enrichment analysis [34] were performed on the intersecting genes, the critical value of FDR < 0.05 was considered statistically significant, and the entry screening criteria were adj. *p* < 0.05 and q-value < 0.05, and the *p*-value correction method was the Benjamini–Hochberg method. Based on the gene expression profile dataset for patients with STAD in TCGA, we performed gene set enrichment analysis (GSEA) [35], and adj. *p* < 0.05 was considered statistically significant.

The STRING (https://string-db.org/) database [36] was used to construct a protein–protein interaction (PPI) network for DEGs in the high- and low-risk groups, with a coefficient of 0.4. PPI results were exported from STRING and visualized using Cytoscape [37], and the “CytoHubba” plugin [38] was used to analyze the bub genes in the PPI network. miRNA–mRNA interaction information was downloaded from the miRTarBas (https://mirtarbase.cuhk.edu.cn/) database [39]. Based on the hub genes identified using the PPI network, an miRNA–mRNA regulatory network was constructed by predicting possible regulated miRNAs.

Immune-related genes were downloaded from a pan-cancer immunogenomic analysis article [40], which included 782 genes and 28 cell types. The degree of immune cell infiltration was analyzed using TCGA-STAD data, and an immune score is obtained for each tumor sample. Tumor Immune Dysfunction and Exclusion (TIDE) [41] (http://tide.dfci.harvard.edu) was used to analyze the treatment response in patients with high- and low-risk scores.

Finally, a nomogram was constructed using the risk score and clinicopathological characteristics significantly associated with OS. Calibration curves were generated to assess nomogram performance.

### Statistical analysis

All data processing and analyses were conducted using R (v4.1.1). To compare two groups of continuous variables, the statistical significance of normally distributed variables was estimated using independent Student’s *t*-tests, and differences between non-normally distributed variables were analyzed using Mann–Whitney U-tests. Chi-square or Fisher’s exact tests were used to compare and analyze statistical significance between two groups of categorical variables. All statistical *p* values were bilateral and statistically significant at *p* < 0.05. Significance labeled as NS, *p* > 0.05; *p < 0.05; **p < 0.01; ***p < 0.001; ****p < 0.0001.

The following packages were used in this study: TCGAbiolinks [42], maftools [43], TCGAmutations, GEOquery [44], GSVA [45], survival, survminer, limma [46], WGCNA [47], Venn, ConsensusClusterPlus [48], survivor, timeROC [49], clusterProfiler [50], ESTIMATE [51], rms, glmnet [52], Pheatmap, and ggplot2.

## Results

### Data pre-processing

**Fig. 2:**
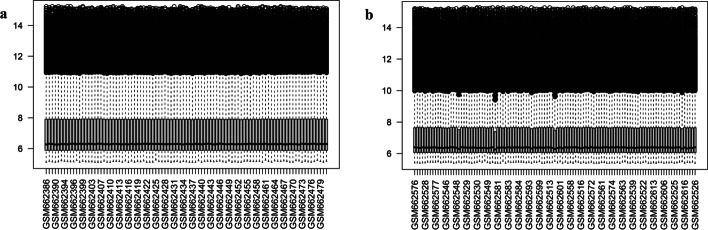
Boxplots of GSE26899 (**a**) and GSE26901 (**b**) expression profile data

First, to analyze the effect of RNA modification on the process of gastric carcinogenesis, we downloaded the gene expression profiles of STAD patients from the TCGA database as the training set and the GSE26899, GSE26901, GSE84437 and GSE62254 gene expression profiles associated with STAD from the GEO database as the validation sets. The box line plots of the gene expression matrices of GSE26899 (Fig. 2a), GSE26901 (Fig. 2b), GSE84437 (Fig. S1a) and GSE62254 (Additional file 2: Fig. S1b) were plotted. The results showed the same expression trends between samples for these datasets, with no intra-group differences, and can be used for subsequent analysis.

### Prognostic analysis of RNAMS in GC and identification of key genes

**Fig. 3:**
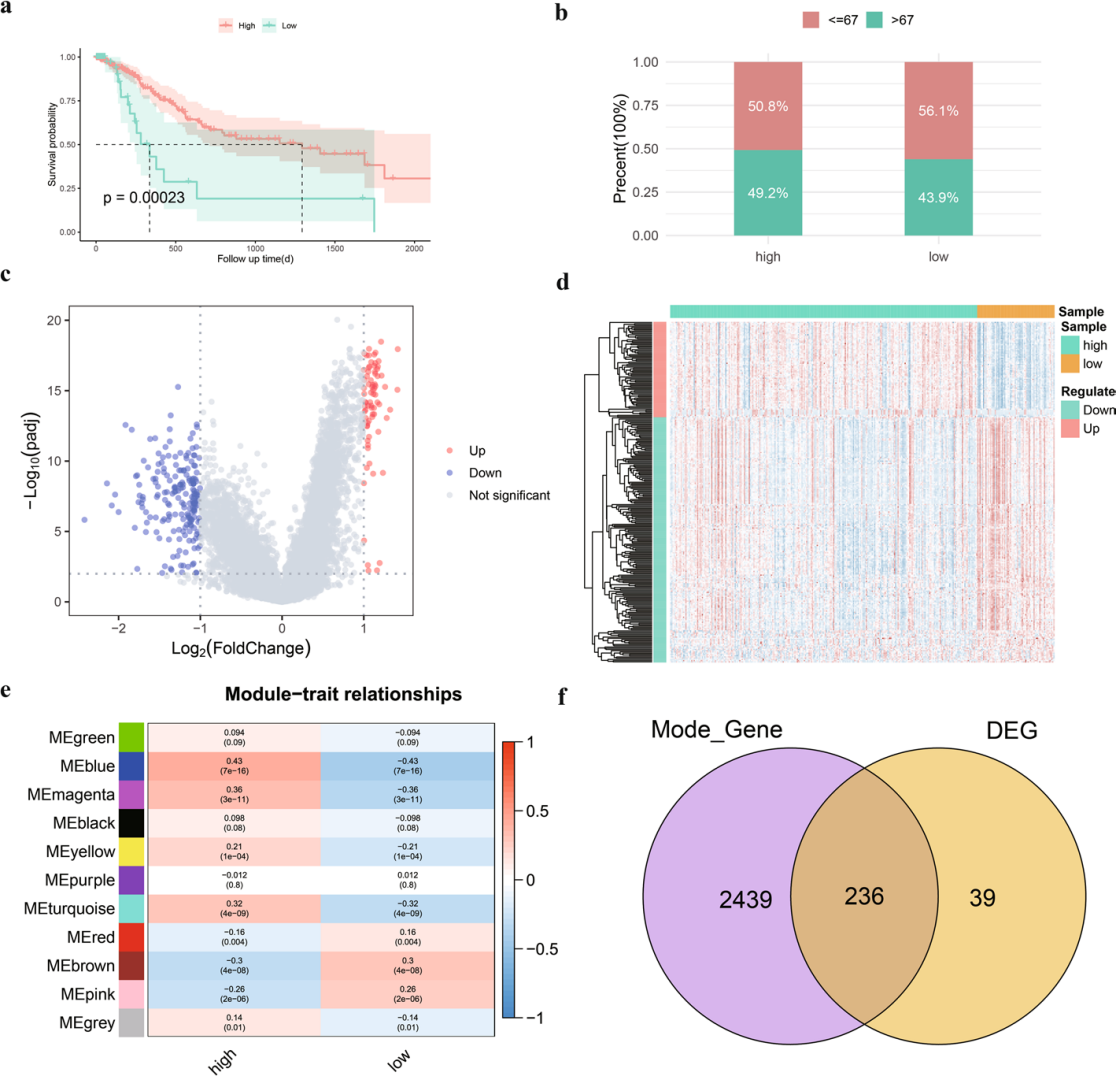
Prognostic analysis of RNA modification score (RNAMS) in gastric cancer and identification of key genes. **a** Survival curves for the high- and low-RNAMS groups; **b** Bar chart of age subgroups in high- and low-RNAMS groups; **c** Differential expression volcano plot of high- and low-RNAMS groups; **d** Heatmap of differentially expressed genes (DEGs) in the high- and low-RNAMS groups; **e** Heatmap of correlation between modules and traits in weighted gene correlation network analysis molecules; **f** Venn diagram of the intersection between DEGs and key gene modules

We calculated the RNAMS for patients with STAD in TCGA to indicate their RNA modification levels. Based on the optimal RNAMS cutoff value (1.207673), TCGA-STAD patients were then divided into high- and low-RNAMS groups. Survival analysis revealed that patients with a high RNAMS had a better prognosis than those with a low RNAMS (Fig. 3a), while clinical analysis showed that patients in the low-RNAMS group had a low age bias and those in the high-RNAMS group had a high age bias (Fig. 3b).

To identify key RNAMRGs in GC, we analyzed DEGs in the high- and low-RNAMS groups (Fig. 3c, d). Of the 275 DEGs identified, 77 were significantly upregulated and 198 were significantly downregulated (Additional file 3: Table S2). Next, we constructed a gene co-expression network to identify biologically significant gene modules as well as genes closely related to RNAMS. Eleven modules were obtained (Fig. 3e) and screened to identify the four modules with the strongest positive and negative correlations with the high-RNAMS group (blue; purple; brown; pink). Intersecting the strongly related genes in the four modules yielded 2,675 module key genes (Additional file 4: Table S3), of which 236 overlapped with the DEGs (Additional file 5: Table S4) and were considered key RNA modification-associated genes in GC (Fig. 3f).

### Analysis of key genes and molecular typing of RNAMS-related genes

**Fig. 4:**
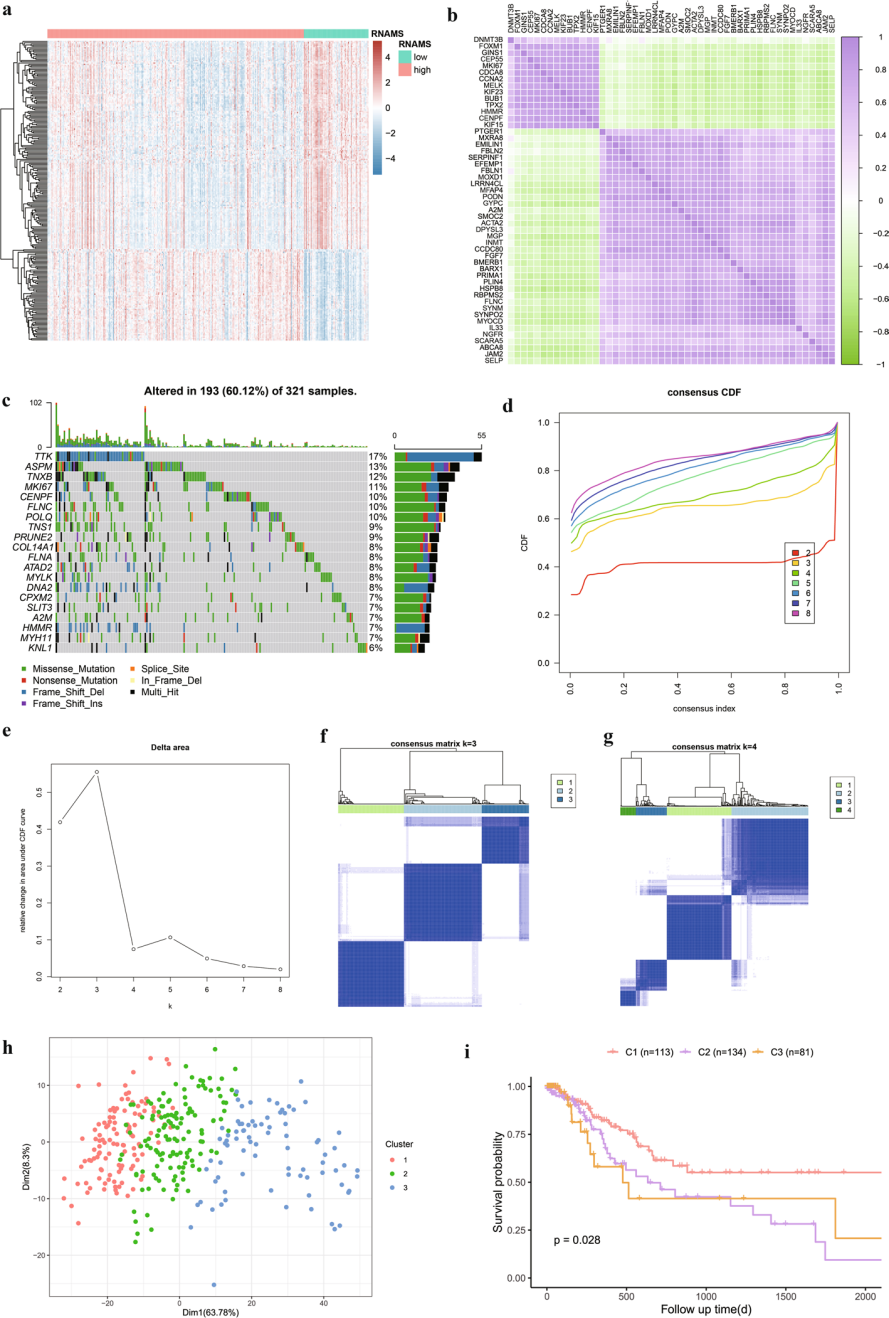
Analysis of key genes and molecular typing of RNA modification score (RNAMS)-related genes. **a** Differential expression heatmap of key genes; **b** Correlation heatmap of key genes; **c** Mutation mapping of RNAMS-associated genes in patients with stomach adenocarcinoma; **d** Consistency cumulative distribution function graph; **e** Scree plot; **f** and **g** Clustering results for *k* = 3 (**f**) and *k* = 4 (**g**); **h** Principal components analysis of clustering results; **i** Survival curves for the three subgroups

To investigate the overall expression of the 236 key genes in patients with STAD, we plotted a heatmap (Fig. 4a) and analyzed their correlation (Additional file 6: Table S5). Fifty genes were randomly selected for visualization (Fig. 4b). Most of the key genes were highly expressed in the low-RNAMS group, and the correlations between genes were generally positive. When we further analyzed the DNA level variation of key genes, we found that single nucleotide polymorphisms (SNPs) were present in key genes in 193 (60.12%) of TCGA-STAD samples (Fig. 4c).

Next, we used the expression profiles of the 236 key genes in all samples to perform unsupervised clustering using a consensus clustering algorithm. The optimal number of clusters was calculated and determined to be three (Fig. 4d–g); therefore, *k* = 3 was used to cluster all samples into three subtypes that were subjected to principal components analysis (Fig. 4h). The three subtypes were well differentiated, and prognostic analysis revealed that survival differed significantly among the three subtypes (Fig. 4i), indicating the accuracy of the clustering results.

### RNAMS risk model construction and evaluation

**Fig. 5:**
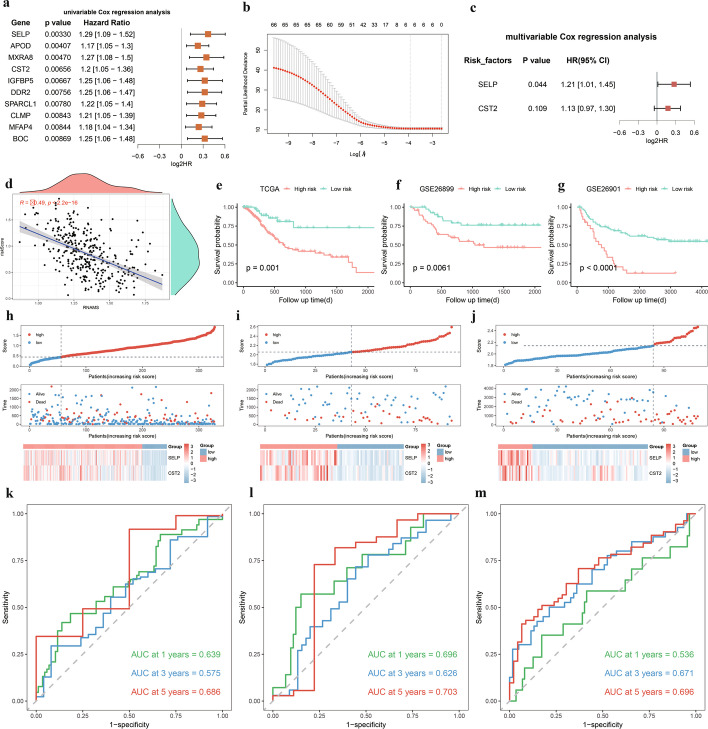
Construction and evaluation of RNA modification score (RNAMS) risk prediction model. **a** Univariate Cox Forest plot for the top 10 genes; **b** Least absolute shrinkage and selection operator regression cross-check lambda results plot; **c** Multivariate Cox Forest plot; **d** Scatter plot of the correlation between risk scores and RNAMS; **e**–**g** Survival curves for The Cancer Genome Atlas (TCGA)-stomach adenocarcinoma (STAD) data (**e**), GSE26899 (**f**), and GSE26901 (**g**); **h**–**j** Risk score distribution for TCGA (**h**), GSE26899 (**i**), and GSE26901 (**j**) expression profiles; **k**–**m** ROC curves for TCGA-STAD (**k**), GSE26899 (**l**), and GSE26901 (**m**)

To quantify the effect of the 236 key genes on prognosis, we combined their expression to construct a risk score model. First, the key genes were screened using univariate Cox regression, and 66 genes were retained for LASSO regression to eliminate covariance (Fig. 5a, Additional file 7: Table S6). After the best lambda values had been determined and crossed by ten-fold validation, six genes (*SELP*, *APOD*, *MXRA8*, *CST2*, *RRAD*, and *GPX3*; Fig. 5b) were subjected to multivariate Cox regression analysis, and the optimal combination was screened using stepwise regression to obtain two genes (*SELP* and *CST2*) for model construction (Fig. 5c). The model scoring formula was as follows:

$$Risk score = \left( {0.189 \times SELP expression} \right) + \left( {0.119 \times CST2 expression} \right)$$

To verify the validity of the model, the correlation between risk score and RNAMS for TCGA patients was plotted (Fig. 5d), and a significant negative correlation was found (cor = -0.49, *p* < 2.2e^−16^). A risk score cutoff value of 0.4448089 was used to classify the training set TCGA-STAD patients into high- and low-risk groups. Survival analysis showed that the low-risk group had significantly better survival than the high-risk group (Fig. 5e). Consequently, patients in the validation sets were classified into high- and low-risk groups based on their respective risk score cutoff values (GSE26899: 2.059207; GSE26901: 2.140648; GSE84437: 2.156697; GSE62254: 1.788483), and survival analysis showed that the low-risk groups had significantly better survival than the high-risk groups (Fig. 5f, g; Additional file 8: Fig. S2a, b). Analyses for risk score distribution, survival status, and characteristic gene expression patterns for TCGA (Fig. 5h), GSE26899 (Fig. 5i), GSE26901 (Fig. 5j), GSE84437 (Additional file 8: Fig. S2c), and GSE62254 (Additional file 8: Fig. S2d) revealed that patients in high-risk groups had worse survival and similar gene expression patterns. Time-dependent ROC analysis of risk scores in the five datasets revealed the AUC values for 1-, 3-, and 5-year OS in TCGA (Fig. 5k), GSE26899 (Fig. 5l), GSE26901 (Fig. 5m), GSE84437 (Additional file 8: Fig. S2e) and GSE62254 (Additional file 8: Fig. S2f) indicating that the risk score could accurately predict the OS of patients with GC.

### Enrichment analysis

**Fig. 6:**
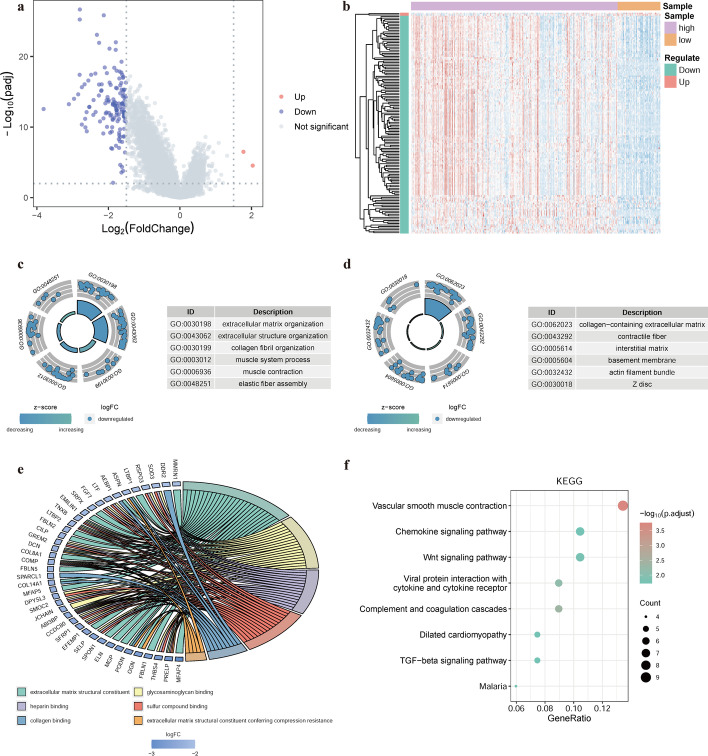
Analysis of differentially expressed genes (DEGs) in high- and low-risk patient groups. **a** Volcano plot of DEGs; **b** Heatmap of DEGs; **c** Top six biological processes; **d** Top six cellular components; **e** Top six molecular functions; **f** Kyoto Encyclopedia of Genes and Genomes pathway enrichment results

To determine the ability of the model to predict cancer development in patients with STAD, we performed differential expression analysis in high- and low-risk patient groups. Of the 144 DEGs identified, 2 were upregulated and 142 were significantly downregulated (Fig. 6a, b). Next, we performed GO and KEGG enrichment analyses to identify biological processes, molecular functions, cellular components, and biological pathways related to the 144 DEGs (Fig. 6c–f, Additional file 9: Tables S7, Additional file 10: Table S8), which included the Wnt signaling pathway and vascular smooth muscle contraction.

**Fig. 7:**
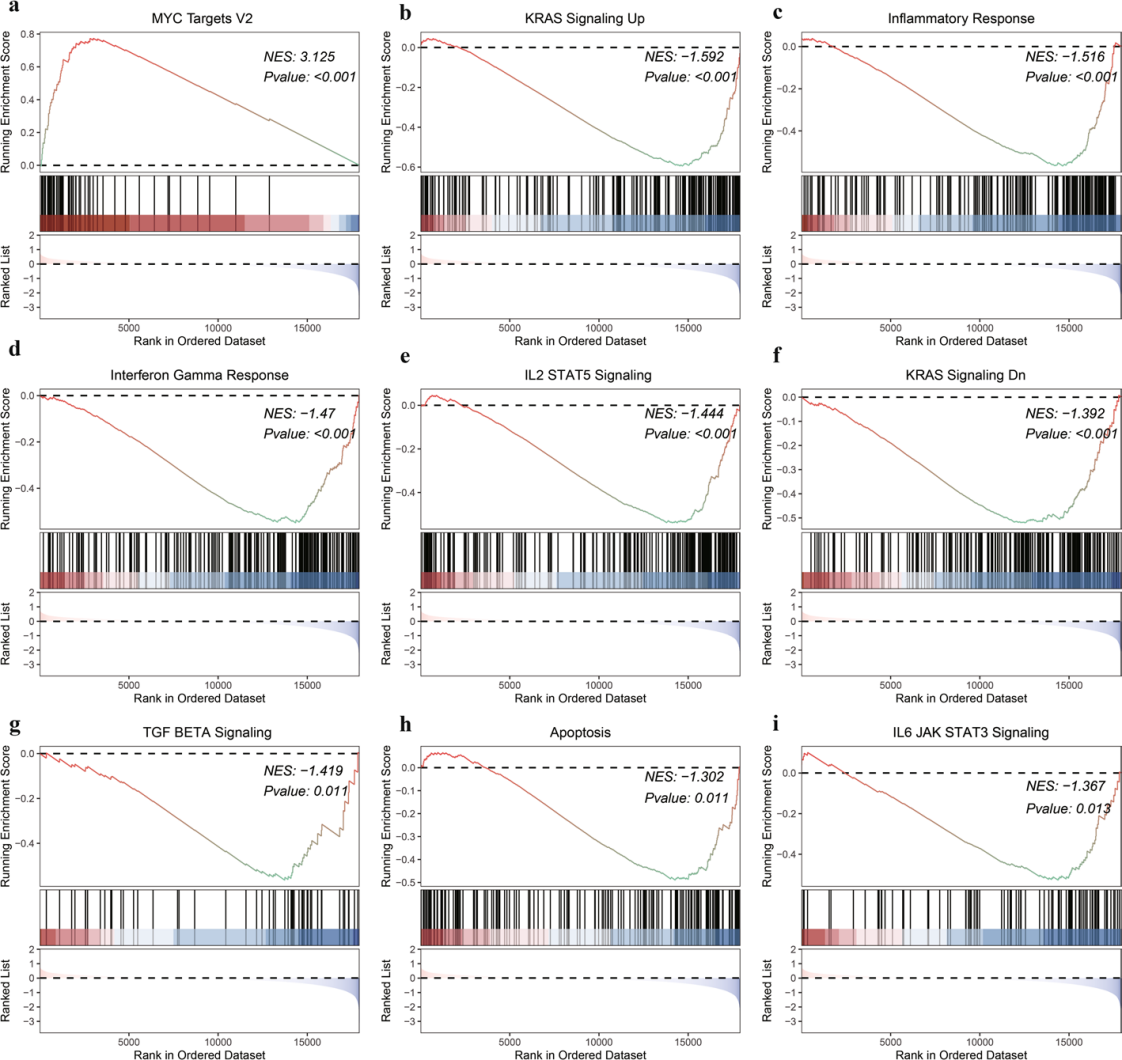
Gene set enrichment analysis. **a** The MYC targets V2 pathway; **b** KRAS signaling up; **c** Inflammatory response; **d** Interferon gamma response; **e** IL2–STAT5 signaling; **f** KRAS signaling dn; **g** TGFβ signaling; **h** Apoptosis; **i** IL6–JAK–STAT3 signaling

To elucidate the effect of gene expression levels on GC, we analyzed the associations between biological processes involved in gene expression in TCGA data using GSEA. The MYC targets V2 pathway (Fig. 7a) was significantly enriched in high-risk patients, whereas KRAS signaling up (Fig. 7b), inflammatory response (Fig. 7c), interferon gamma response (Fig. 7d), IL2–STAT5 signaling (Fig. 7e), KRAS signaling dn (Fig. 7f), TGFβ signaling (Fig. 7g), apoptosis (Fig. 7h), and IL6–JAK–STAT3 signaling (Fig. 7i) were significantly enriched in low-risk patients (Additional file 11: Table S9).

### Construction of PPI and related regulatory networks

**Fig. 8:**
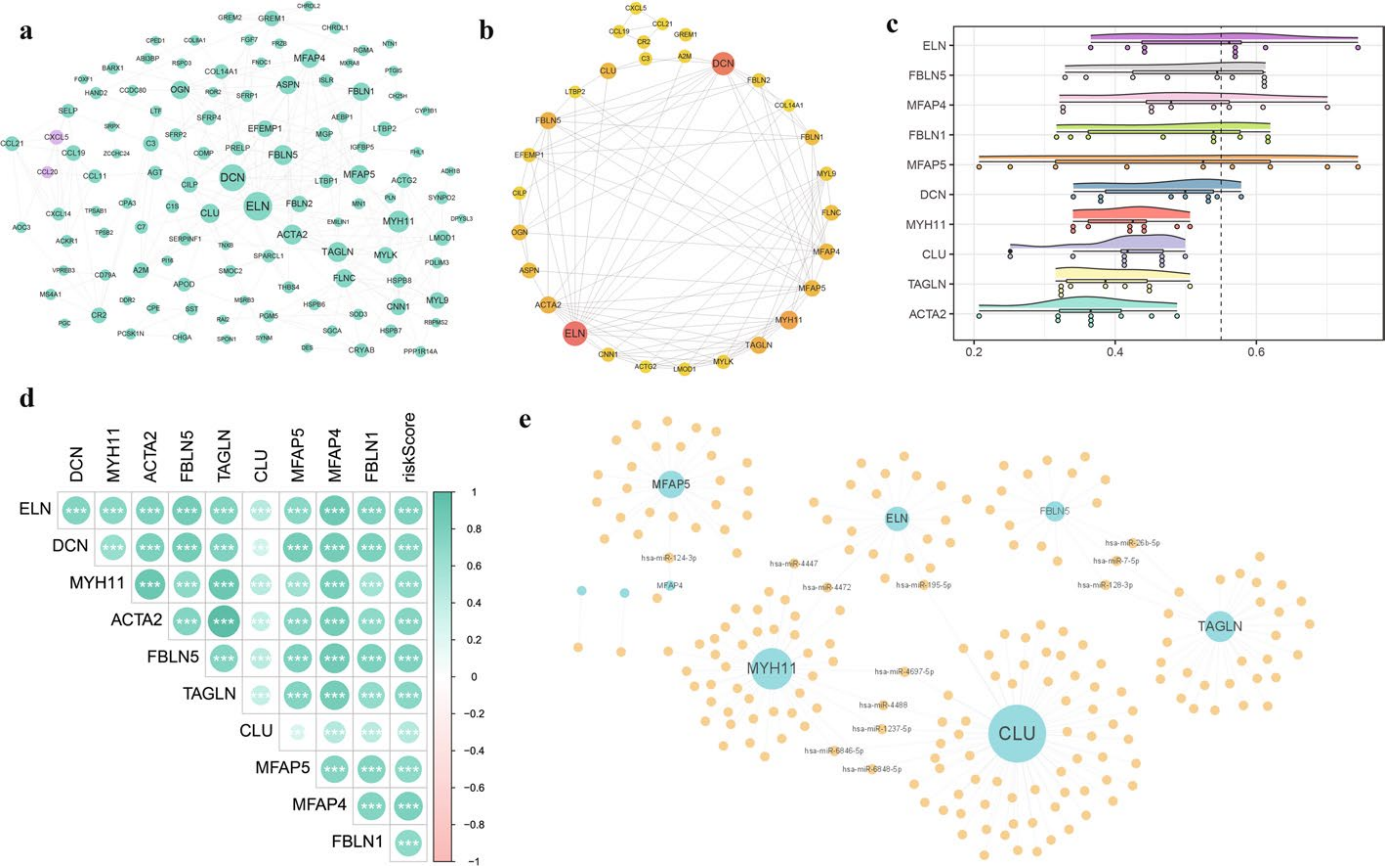
Construction of protein–protein interaction (PPI) and related regulatory networks. **a** PPI network of differentially expressed genes; **b** Maximal Clique Centrality network of top 10 nodes. Darker color indicates more important nodes; **c** Hub gene importance raincloud plot; **d** Heatmap of correlations between hub genes and risk scores; **e** miRNA–mRNA regulatory network constructed using hub genes. Node size indicates its connectivity in the network

To analyze the protein interactions among the 144 DEGs, we constructed a PPI network (Fig. 8a). The top 10 hub genes (*ELN*, *DCN*, *MYH11*, *ACTA2*, *FBLN5*, *TAGLN*, *CLU*, *MFAP5*, *MFAP4*, and *FBLN1*) were obtained based on local node density, with a darker color indicating node importance (Fig. 8b). Functional similarity (Friends) analysis revealed that *ELN* was an important hub gene (Fig. 8c), while correlation analysis indicated significant co-expression patterns between hub genes and risk scores, all of which correlated positively (Fig. 8d). Based on miRNA–mRNA interaction information downloaded from the miRTarBase database, an miRNA–mRNA regulatory network was constructed using the hub genes obtained from the PPI network (Fig. 8e), which contained a total of 202 miRNAs and 9 mRNAs worthy of further study.

### Immune cell infiltration analysis

**Fig. 9:**
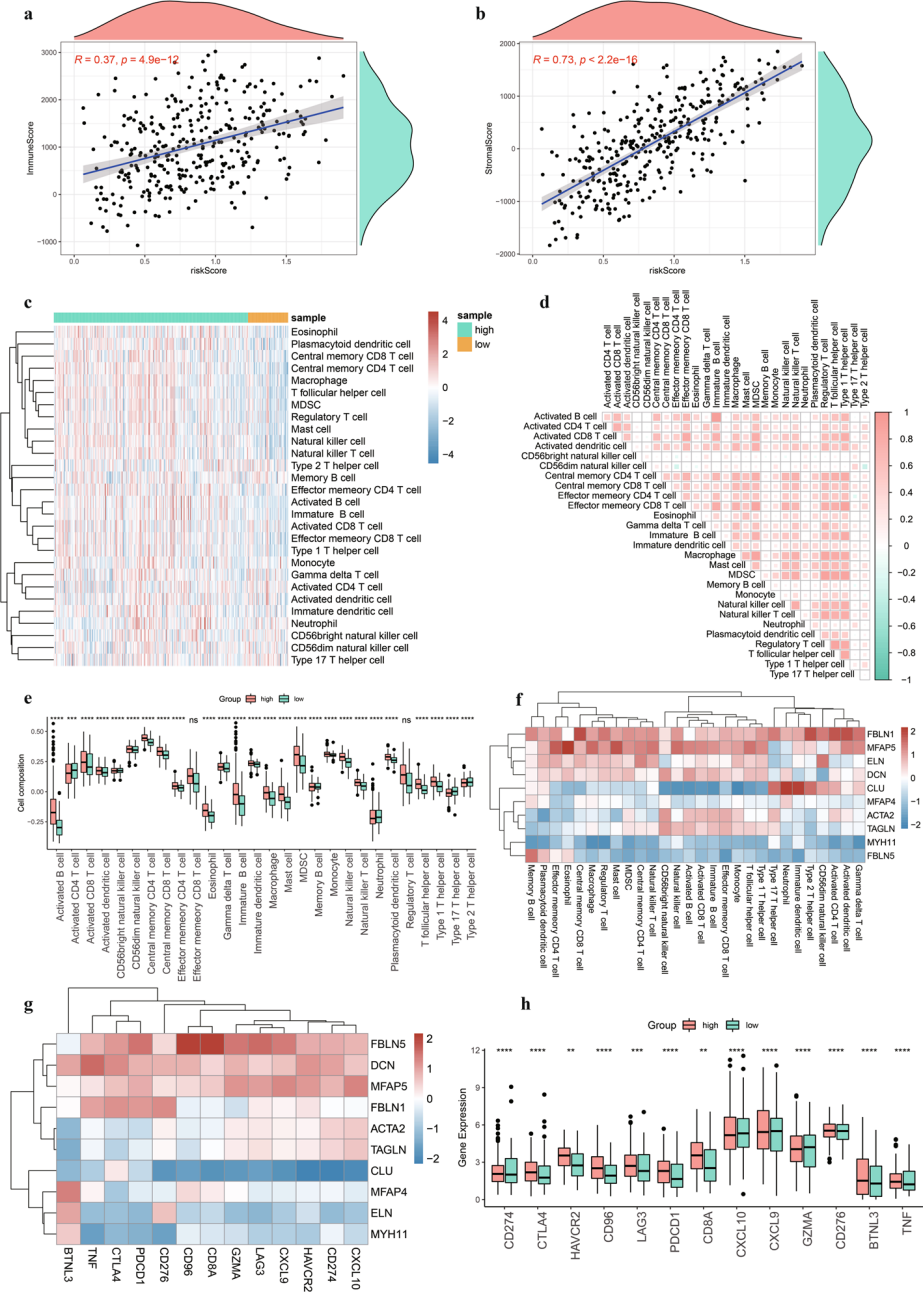
Immune cell infiltration analysis. **a** Scatter plot of correlation between risk score and immune score; **b** Scatter plot of correlation between risk score and stromal score; **c** Heatmap of immune infiltration; **d** Heatmap of immune cell correlations; **e** Boxplot of differences in immune cell infiltration in high- and low-risk groups. **f** Heatmap of correlations between hub genes and immune cell infiltration; **g** Heatmap of correlations between hub genes and immune genes; **h** Boxplot of differences in immune genes in high- and low-risk groups

To evaluate the effect of the risk score on the overall immune characteristics and immune cell infiltration levels of patients with STAD, we calculated immune and stromal scores for each patient. A significant positive correlation was observed between risk score and both the immune score (cor = 0.37, *p* = 4.9e^−12^, Fig. 9a) and stromal score (cor = 0.73, *p* < 2.2e^−16^, Fig. 9b). Immune cell infiltration scores in TCGA were displayed using a heatmap to further examine immune cell expression (Fig. 9c). Most immune cells correlated positively with one another (Fig. 9d), with > 90% of immune cells in both high- and low-risk groups showing a significant difference and most immune cells displaying significantly higher infiltration in the high-risk group than in the low-risk group (Fig. 9e).

Next, we plotted the correlation heatmap between hub genes and immune cells (Fig. 9f) and between hub genes and immune genes (Fig. 9g) to determine the effect of hub genes on immune function in patients with STAD. Differential boxplots revealed that the expression of all immune genes differed significantly between the high- and low-risk groups (Fig. 9h).

### Effect of risk score on genomic changes in patients with STAD

**Fig. 10:**
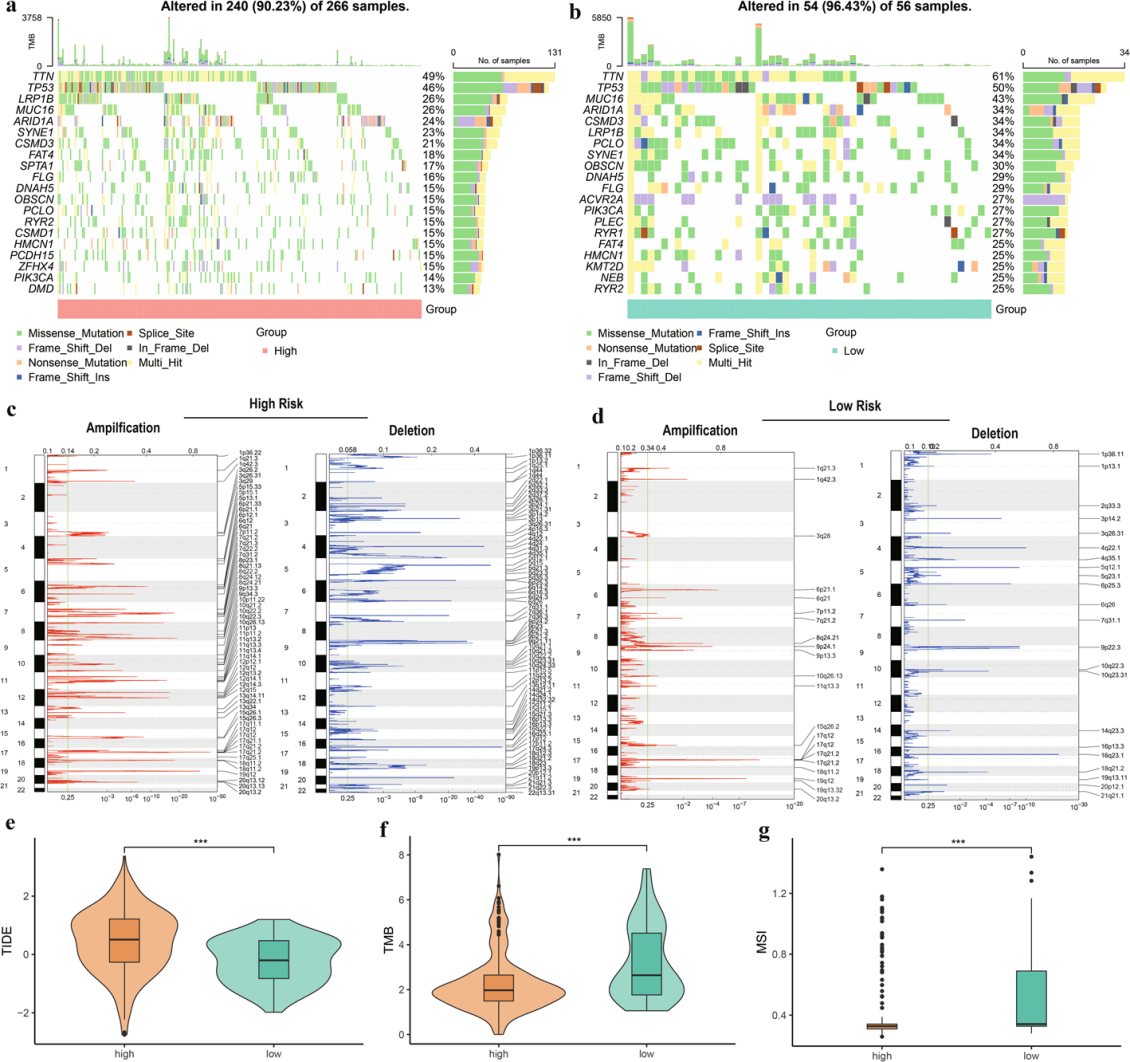
Effect of risk score on genomic changes in patients with stomach adenocarcinoma. **a** and **b** Mutation profiles of common tumorigenic driver genes in high-risk group (**a**) and low-risk group (**b**); **c** and **d** Changes in copy number levels of genes in the high-risk group (**c**) and low-risk group (**d**); **e**–**g** Boxplots of the difference in Tumor Immune Dysfunction and Exclusion (TIDE) (**e**), tumor mutational burden (TMB) (**f**), and microsatellite instability (MSI) (**g**) in high- and low-risk groups

To determine the effect of the risk score on genetic variation in patients with STAD, we analyzed changes in SNPs and CNVs. The overall level of single nucleotide mutations in common tumorigenesis driver genes was lower in the high-risk group than in the low-risk group (Fig. 10a, b), whereas the frequency of CNVs was significantly increased in all high-risk patients, particularly for copy number deletions (Fig. 10c, d). By calculating and plotting the TIDE, TMB, and MSI, we found that TIDE scores were significantly higher in the high-risk group than in the low-risk group (*p* = 2.6e^−06^, Fig. 10e), whereas TMB (*p* = 1.8e^−05^, Fig. 10f) and MSI (*p* = 6.7e^−05^, Fig. 10g) were significantly lower in the high-risk group than in the low-risk group. Together, these results indicate that the model risk score can be a potential indicator for evaluating the efficacy of immunotherapy in patients with GC.

### Construction of a clinical predictive nomogram

**Fig. 11:**
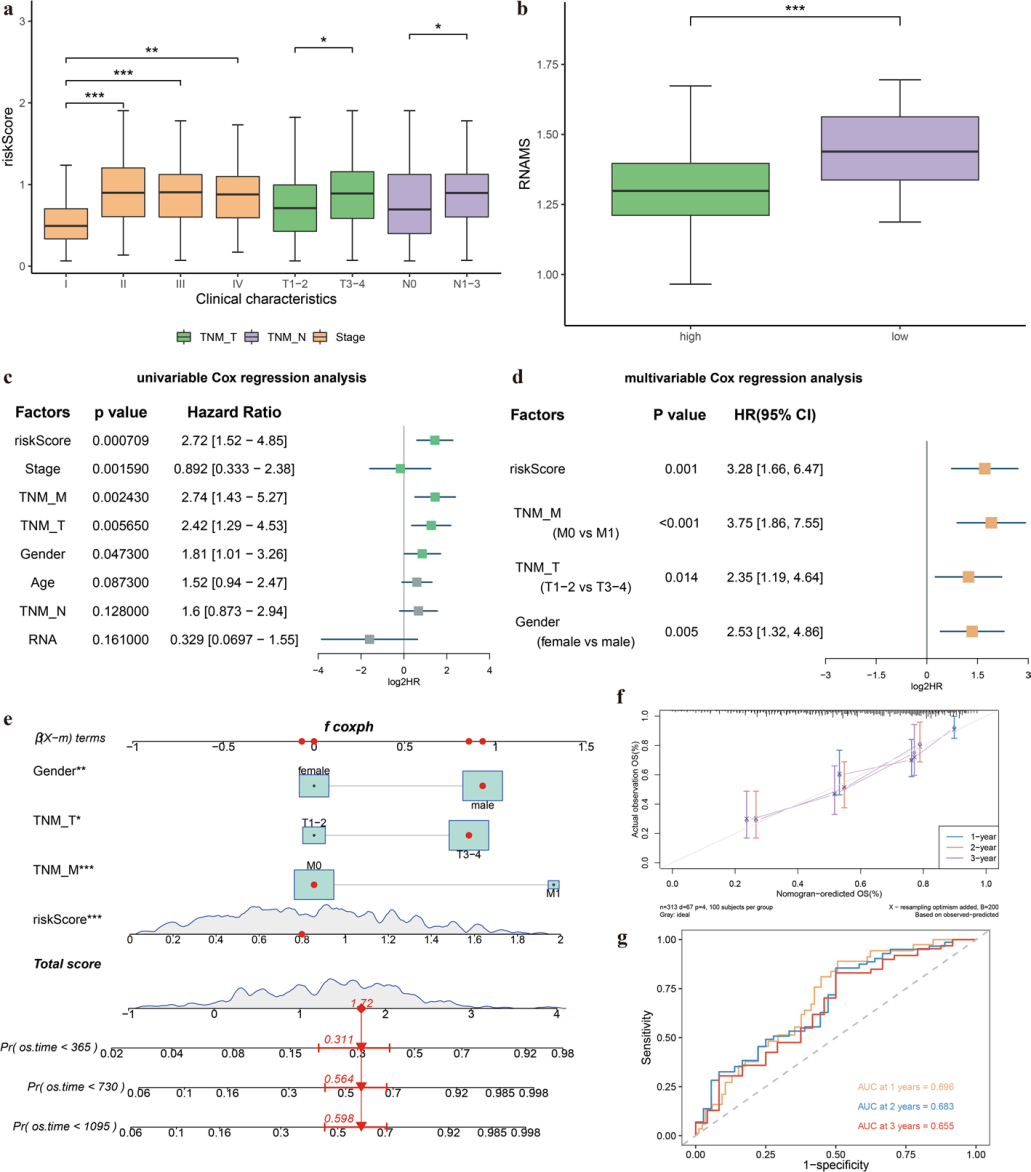
Construction of a nomogram based on the risk score. **a** Boxplot of differences in risk scores across clinical characteristics; **b** Boxplot of differences between risk scores and RNA modification scores; **c** Univariate Cox regression Forest plot; **d** Multivariate Cox regression Forest plot; **e** Nomogram predicting the 1-, 2-, and 3-year overall survival status of patients with gastric cancer; **f** Nomogram calibration curve. **g** 1-, 2-, and 3-year receiver operating characteristic curves for the nomogram. AUC: area under curve

Finally, we generated boxplots to visualize the differences between risk score and clinical characteristics. Risk score differed significantly with pathological stage, T stage, and N stage (Fig. 11a), and the RNAMS was significantly lower in the high-risk group than in the low-risk group (*p* = 1.5e^−08^, Fig. 11b). To determine whether the risk score and clinicopathological characteristics were independent prognostic factors, we performed regression analyses. Univariate Cox regression analysis showed that risk score (*p* < 0.001), pathological stage (*p* = 0.00159), M stage (*p* = 0.00243), T stage (*p* = 0.00565), and gender (*p* = 0.0473) were all associated with OS (Fig. 11c, Table 2), and multivariate Cox regression analysis of these significant factors revealed that risk score (*p* = 0.001), M stage (*p* < 0.001), T stage (*p* = 0.014), and gender (*p* = 0.005) were significantly associated with OS (Fig. 11d, Table 3). Therefore, we combined the risk score with these three clinicopathological characteristics to construct a predictive nomogram to assess the OS of patients with STAD (Fig. 11e). Calibration curves revealed good uniformity between the OS estimates of the nomograph for 1-, 2- and 3-year OS of patients and the actual observed values (Fig. 11f), suggesting that the nomogram predictions were accurate.

**Table 2:** Univariate Cox regression analysis results

	Hazard ratio	Lower 95% CI	Upper 95% CI	*p*
Risk score	2.72	1.52	4.85	0.000709
Stage	0.892	0.333	2.38	0.00159
TNM_M	2.74	1.43	5.27	0.00243
TNM_T	2.42	1.29	4.53	0.00565
Gender	1.81	1.01	3.26	0.0473
Age	1.52	0.94	2.47	0.0873
TNM_N	1.6	0.873	2.94	0.128
RNA	0.329	0.0697	1.55	0.161

*CI* Confidence level

**Table 3:** Multivariate Cox regression analysis results

	Exp (coefficient)	Lower 95% CI	Upper 95% CI	*p*
Risk score	3.28	1.66	6.47	0.001
TNM_M (M0 vs. M1)	3.75	1.86	7.55	< 0.001
TNM_T (T1–2 vs. T3–4)	2.35	1.19	4.64	0.014
Gender (female vs. male)	2.53	1.32	4.86	0.005

*CI* Confidence level

The ROC curves of the prognostic discrimination of patients at 1 year, 2 years and 3 years of the nomogram were plotted (Fig. 11g), and the results showed that the AUC values of the nomogram for predicting the prognosis of patients at 1 year, 2 years and 3 years were 0.696, 0.683 and 0.655, respectively, indicating that the nomogram has good predictive ability.

## Discussion

Although the incidence and mortality rates of GC have declined in the past 5 decades [53–55], GC remains the third leading cause of cancer-related deaths [56]. Cancer cells are characterized by genomic instability, which favors mutation accumulation and increased tumor heterogeneity [57–59]. RNA modifications have recently emerged as key post-transcriptional regulators of gene expression and are thought to be associated with various diseases, including cancer [60]. However, the intrinsic relationship between RNA modifications and GC progression remains unknown. In this study, we analyzed the relationship between RNAMRGs and GC using bioinformatics analyses and statistical methods and constructed a prognostic model.

First, we scored each TCGA sample and grouped the samples according to their RNAMS and found that patients with high RNA modification correlation had a worse prognosis. Dynamic RNA modification events can promote tumor progression by promoting tumor cell proliferation or regulating invasive and metastatic potential, resulting in a poor prognosis, consistent with our findings. Therefore, we explored hub RNAMRGs and established a prognostic prediction model for patients with GC using selectin P (*SELP*) and cystatin SA (*CST2*). *SELP* encodes a protein stored on platelets and endothelial cells that is redistributed to the plasma membrane during platelet activation and degranulation and mediates the interactions between activated endothelial cells or platelets and leukocytes [61]. Coxsackievirus B 1 and 3 interact with human platelets to trigger *SELP* expression [62], and *SELP* has predictive value in various cancers [63, 64]; however, the relationship between *SELP* and RNA modification in GC carcinogenesis and development remains unclear. *CST2* overexpression enhances the growth, migration, and invasion of GC cells by regulating the epithelial–mesenchymal transition and TGF-β1 signaling pathways [65]. According to the formula of our predictive model, higher *SELP* and *CST2* expression were associated with a higher risk score, and survival analysis further indicated that the high-risk group had a worse prognosis. The high predictive efficacy of the model was further validated using GEO datasets. Taken together, these findings are consistent with the hypothesis that *SELP* and *CST2* may act as cancer progression-promoting genes in various tumors.

Furthermore, enrichment analysis revealed the importance of the Wnt signaling pathway, which plays a key role in tumorigenesis [66, 67]. While constructing the PPI network, we identified *ELN* as an important hub gene. *ELN* encodes one of the two components of elastic fibers that form part of the extracellular matrix and confer elasticity to organs and tissues. Abnormal ELN levels have been observed in many fibrotic diseases, including kidney [68], lung [69], and liver fibrosis [70], and ELN accumulation is associated with the development of hepatocellular carcinoma [71]. *ELN* has also been shown to regulate tumor development and the tumor microenvironment (TME) in colorectal cancer [72]. Therefore, studying the mechanism of *ELN* and RNA modification in GC carcinogenesis is essential. miRNAs are epigenetic regulators that affect the levels of proteins encoded by target mRNAs without modifying gene sequences and are themselves regulated by epigenetic mechanisms [73]. Reciprocal relationships between miRNAs and epigenetic regulation form miRNA-epigenetic feedback loops that can regulate cellular processes such as cell proliferation [74], apoptosis [75], and differentiation [76]. To understand the regulatory relationship between the hub genes (e.g., *ELN*) and miRNAs, we constructed an miRNA–mRNA regulatory network to explore whether miRNAs and mRNAs are associated with GC development.

Recently, breakthroughs have been made in immunotherapy for cancers [77], including GC [78, 79]; however, not all patients with GC benefit from immunotherapy, possibly owing to the TME [80]. Here, we found that immune cell infiltration was increased in the high-risk group. *FBLN1* was highly expressed in most immune cells and correlated positively with the majority of immune genes, whereas *FBLN5* was expressed at low levels in most immune cells. FBLN1 and FBLN5 are components of extracellular matrix fibronectin, and ectopic FBLN1 expression inhibits the growth of GC cells by inducing apoptosis [81]. Additionally, FBLN5 is a potential indicator of therapeutic efficacy in patients with hepatocellular carcinoma [82]. Thus, *FBLN1* and *FBLN5* may be potential immunotherapeutic targets for treating GC. A comparison of the high- and low-risk groups revealed that all immune genes exhibited significantly different expression patterns, suggesting that our predictive model is a potential indicator for immunotherapy in patients with GC.

We also found that overall mutation levels for common tumor driver genes were lower in the high-risk group than in the low-risk group, while CNVs, particularly due to deletion, were significantly increased in all patients in the high-risk group. TIDE, TMB, and MSI results further suggested that our model could predict the effect of immune checkpoint inhibition therapy in patients with GC. We therefore combined the model with clinicopathological features to construct a nomogram to assess the clinical prognosis of GC. Despite these promising findings, our study had some limitations. First, the model has not yet been applied in a clinical setting, and its actual predictive accuracy remains unknown. Second, the value and mechanism of action of the hub RNAMRGs identified in this study have not been experimentally verified in patients with GC. To overcome these limitations, we will apply the nomogram in future clinical work and conduct further experiments to explore the mechanism of key RNA modifications in the development of GC.

In conclusion, the RNAMRG risk prediction model developed in this study could effectively predict the overall survival of patients with GC. Thus, the model risk score can be used to study GC carcinogenesis and developing targeted therapies for GC.

## Supplementary Information

**Additional file 1. Table S1:** RNA modification-related genes.

**Additional file 2. Figure S1:** Boxplots of GSE84437 (a) and GSE62254 (b) expression profile data.

**Additional file 3. Table S2:** Differentially expressed genes in the high- and low-RNA modification score groups.

**Additional file 4. Table S3:** Key gene modules.

**Additional file 5. Table S4:** Key genes.

**Additional file 6. Table S5:** Correlation among all key genes.

**Additional file 7. Table S6:** Univariate Cox regression analysis results for all key genes.

**Additional file 8. Figure S2:** Evaluation of RNA modification score (RNAMS) risk prediction model. a–b Survival curves for GSE84437 (a) and GSE62254 (b); c–d Risk score distribution for GSE84437 (i) and GSE62254 (j) expression profiles; e–f ROC curves for GSE84437 (e) and GSE26901 (f).

**Additional file 9. Table S7:** Gene Ontology enrichment analysis results.

**Additional file 10. Table S8:** Kyoto Encyclopedia of Genes and Genomes enrichment analysis results.

**Additional file 11. Table S9:** Gene set enrichment analysis results.

## Data Availability

The datasets analyzed during the current study are available in The Cancer Genome Atlas (https://portal.gdc.cancer.gov) and the Gene Expression Omnibus (https://www.ncbi.nlm.nih.gov/geo/query/acc.cgi?acc=GSE26899, https://www.ncbi.nlm.nih.gov/geo/query/acc.cgi?acc=GSE26901, https://www.ncbi.nlm.nih.gov/geo/query/acc.cgi?acc=GSE84437 and https://www.ncbi.nlm.nih.gov/geo/query/acc.cgi?acc=GSE62254).All data used in this study are available from public databases and can be provided by the authors upon reasonable request.
