# Supplementary figures and images for: Construction of an RNA modification-related gene predictive model associated with prognosis and immunity in gastric cancer
Source: BMC Bioinformatics. 2023 Apr 15;24:147. doi: 10.1186/s12859-023-05283-3 (PMC10105968; doi:10.1186/s12859-023-05283-3)

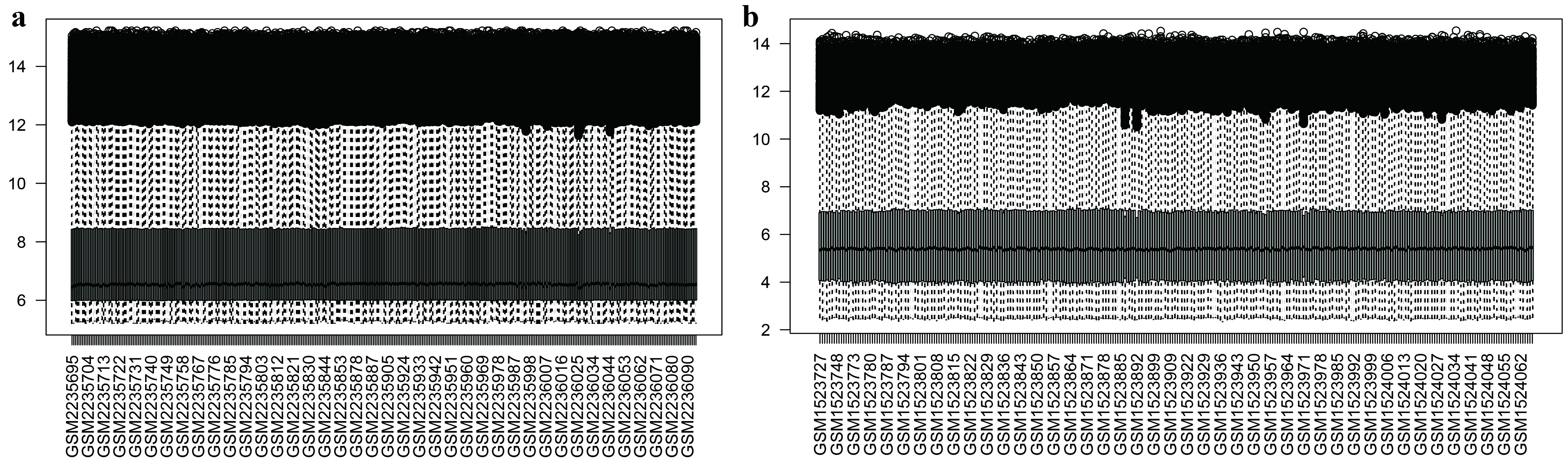

Supplement: Supplementary file 2 — Additional file 2. Figure S1: Boxplots of GSE84437 (a) and GSE62254 (b) expression profile data. [file 12859_2023_5283_MOESM2_ESM.tif]
